# The endothelial plasma membrane lipidome and its remodeling under hyperglycemia: an exploratory study

**DOI:** 10.3389/fmolb.2025.1701375

**Published:** 2026-02-16

**Authors:** Ana Reis, Yahya Sohrabi, Lorena Diaz-Sanchez, Ana Rita Dias Araújo, Merle Leffers, Bruno Antonny, Alisa Rudnitskaya, Rui Vitorino, Irundika H. K. Dias

**Affiliations:** 1 REQUIMTE/LAQV, Departamento de Química e Bioquímica, Faculdade de Ciências, Universidade do Porto, Rua do Campo Alegre, Porto, Portugal; 2 Department of Cardiology I, Coronary, Peripheral Vascular Disease and Heart Failure, University Hospital Münster, University of Münster, Münster, Germany; 3 Aston Medical School, College of Health and Life Sciences, Aston University, Birmingham, United Kingdom; 4 Université Côte d’Azur, CNRS and Inserm, Institut de Pharmacologie Moléculaire et Cellulaire, Valbonne, France; 5 CESAM/Department of Chemistry, University of Aveiro, Aveiro, Portugal; 6 iBIMED, Institute of Biomedicine, Department of Medical Sciences, Aveiro, Portugal

**Keywords:** cholesterol, giant plasma membrane vesicles, human umbilical vein endothelial cells, oxidized phospholipids, oxysterols, shotgun mass spectrometry

## Abstract

**Introduction:**

At the interface between blood and blood vessels, the endothelial plasma membrane is the first point of contact to external stimuli, triggering the cascade of intracellular events responsible for proper vascular function. However, the endothelial plasma membrane lipidome and its remodeling in pathological conditions remain largely unknown.

**Methods:**

To address this gap, we present a comprehensive lipidomic analysis of cell-derived giant plasma membrane vesicles isolated from primary human umbilical vein endothelial cells cultured *in vitro* under normoglycemic conditions and their lipid remodeling in adaptation to hyperglycemia.

**Results:**

Using targeted mass spectrometry-based strategies, 251 lipids and 13 oxidized lipids from 20 subclasses were identified and quantified. Cholesterol accounted for almost half (45 mol%) of the membrane’s composition. In adaptation to hyperglycemia, the noticeable decrease in the total phospholipids extracted resulted in an increased cholesterol-to-phospholipid (Chol/PL) ratio, which is consistent with increased membrane stiffening. Several other lipid subclasses, namely, lysolipids, phosphatidylcholines, aminophospholipids, polyunsaturated sphingomyelins, and other polyunsaturated phospholipids, showed a decreasing trend. Oxysterols displayed a shift toward the predominance of enzymatic (tail-oxidized) in hyperglycemia, whereas truncated oxidized phosphatidylcholines (oxPC) with a terminal aldehyde moiety exhibited a decreasing trend, suggesting the formation of lipid–protein cross-linking modification.

**Discussion:**

The hyperglycemia-induced alterations provide insights into the endothelial membrane lipid environment and the biophysical dynamics that are likely to deregulate protein–lipid interactions involved in sugar and lipid metabolism. The high amount of Chol found in our work serves as the basis for future *in silico* simulations crucial for drug design and drug response evaluation.

## 1 Introduction

Soft drinks and sugary snacks are routinely consumed in fast-paced modern lives, leading to a sharp increase in sugar intake and contributing to the escalating incidence of type 2 diabetes mellitus (T2DM) in the worldwide population. Over the past 3 decades, the number of adults diagnosed with diabetes has quadrupled, reaching 537 million people (Ong et al., 2023), and many remain undiagnosed. Even more concerning is the rising incidence of diabetes among children and young adults (Wu et al., 2022). The excess of glucose in the bloodstream (hyperglycemia) is particularly detrimental to the layer of cells that line blood vessels (endothelium), where the associated oxidative response and dyslipidemia trigger the secretion of pro-inflammatory cytokines and other vascular mediators, leading to the onset of endothelial dysfunction (Fiorello et al., 2020; Tabit et al., 2010). The hyperglycemia-induced endothelial dysfunction contributes to complications in the microvasculature (e.g., heart disease, stroke, retinopathy, nephropathy, and neuropathy) and ultimately to the increased cerebro- and cardio-vascular morbidity and mortality. In an aging population, the economic burden of T2DM and associated complications poses a major challenge to the sustainability of healthcare systems.

To date, much research has focused on deciphering the changes to the circulating lipid cargo (dyslipidemia) in T2DM that lead to endothelial dysfunction and lipid accumulation in the vessel wall (Alshehry et al., 2016; Razquin et al., 2018; Chew et al., 2019; Meikle et al., 2014). However, endothelial dysfunction is a complex interplay of events of circulating extracellular stimuli (excess glucose and atherogenic lipoproteins) on the surface of endothelial cells (ECs). This interaction triggers a complex cross-talk and intracellular response, causing ECs to alter their morphology, protein expression, and lipid composition in response to short-term (mechanical stress) and long-term (age and disease) external stimuli (Chen et al., 2013; Hashimoto et al., 1999; Sahni et al., 2023; Venturini et al., 2019). In fact, previous fluorescence studies have shown that EC were able to somehow “sense” alterations to the surrounding hemodynamic environment, changing the lipid composition of the plasma membrane (PM) lying at the interface between blood and blood vessels and the first point of contact of external stimuli (Yamamoto and Ando, 2013; Yamamoto and Anto, 2015). Despite the key role of the endothelial PM in this complex cross-talk, very little is known about the lipid (micro) environment in which membrane proteins (receptors) are embedded. Previous large-scale lipidomic studies on human EC (Hirata et al., 2021; Hong et al., 2024; Yang et al., 2011; Qin et al., 2024) focused on whole cell extracts, not the plasma membrane, and excluded quantification of cholesterol (Chol) in their analysis strategy. Recently, work conducted on epithelial-like cell lines revealed that chemically induced plasma membrane vesicles (e.g., GPMVs) display a unique phospholipidomic signature that is cell-type-specific (Symons et al., 2021), prompting further studies to decipher the lipid composition of PM in human primary EC and any changes induced by hyperglycemia.

Within the framework of Working Group 4 of the COST Action EpiLipidNET (CA 19105), committed to advance the integration of (epi-)lipidomics in model systems (https://www.epilipid.net/interest-group-on-endothelial-plasma-membrane-lipidome-endotheliome/), we describe the exploratory study of the endothelial plasma membrane lipidome prepared from primary human umbilical vein endothelial cells (HUVECs) and the lipid remodeling undergone by endothelial PM in adaptation to *in vitro* glucotoxic conditions (hyperglycemia).

## 2 Materials and methods

### 2.1 Reagents

Synthetic lipids and non-naturally occurring lipid species were purchased from Avanti Polar Lipids (Alabaster, AL, United States) except for deuterated free cholesterol (FC[d7]) purchased from Cambridge Isotope Laboratories (Andover, MA, United States) with isotope purity >98%. Lipid standards were dissolved in chloroform and added to endothelial GPMVs prior to extraction and used for quantification by shotgun lipidomics, as described earlier (Heimerl et al., 2023). These are as follows: FC[d7], CE 17:0, CE 22:0, SM 18:1; O2/12:0, Cer 18:1; O2/14:0, Cer 18:1; O2/17:0, HexCer 18:1; O2/12:0, HexCer 18:1; O2/17:0, LPC 13:0/0:0, LPC 19:0/0:0, LPE 13:0/0:0, PC 14:0/14:0, PC 22:0/22:0, PE 14:0/14:0, PE 20:0/20:0, PI 17:0/17:0, PS 14:0/14:0, PS 20:0/20:0, PA 14:0/14:0, PA 16:0/16:0, PG 14:0/14:0, CL 14:0/14:0/14:0/14:0, DG 14:0/14:0/0:0, DG 20:0/20:0/0:0, TG 17:0/17:0/17:0, and TG 19:0/19:0/19:0.

Lipid standards for high-performance thin-layer chromatography (HPTLC) analysis were purchased from Sigma and Avanti Polar Lipids, as follows: L-α-lysophosphatidylcholine (LPC, 830071), sphingomyelin (SM, 860062), L-α-phosphatidylcholine (PC, 840051), L-α-phosphatidylserine (PS, 840032), L-α-phosphatidylinositol (PI, 840042), cardiolipin (CL, 840012), L-α-phosphatidic acid (PA, 840101), L-α-phosphatidylethanolamine (PE, 840026), L-α-phosphatidylglycerol (PG, 841138), C24 lactosyl(ß)-ceramide (d18:1/24:0) (LacCer/Hex2Cer, 860577), mono-sulfo galactosyl(ß)-ceramide (MSGCer, 860572), glucosyl(ß)-ceramide (GlucCer/HexCer, 860569), ceramide (860052, Cer), 5-cholesten-3β-ol (Chol, C8667), cholest-5-en-3ß-yl heptadecanoate (CholE, 700186), cholesteryl formate (CholF, S448532), arachidonic acid (FFA, A3611), 1-oleoyl-rac-glycerol (MG, 330724), 1,3-di(cis-9-octadecenoyl)-glycerol (DG1.3, D3627), 1,2-dipalmitoyl-sn-glycerol (DG1.2, 800816), and 1,2,3-tri-(9Z-octadecenoyl)-glycerol (TG, T7140).

Synthetic deuterated oxysterols (24OHC-d7, 25OHC-d6, 27OHC-d6, 7βOHC-d7, and 7-ketoC-d5) were used as internal standards. Authentic oxysterols (24(S)-hydroxycholesterol, 26-hydroxycholesterol, 25-hydroxycholesterol, and 7β-hydroxycholesterol) were used for external calibration curves. Oxidized phosphatidylcholines (1-palmitoyl-2-(5′-oxo-valeroyl)-sn-glycero-3-phosphochatidylline (POVPC); 1-palmitoyl-2-(9′-oxo-nonanoyl)-sn-glycero-3-phosphatidylcholine (PONPC); 1-palmitoyl-2-glutaryl-sn-glycero-3-phosphochatidylline (PGPC); 1-palmitoyl-2-azelaoyl-sn-glycero-3-phosphatidylcholine (PAzPC); 1-stearoyl-2-(5′-oxo-valeroyl)-sn-glycero-3-phosphochatidylline (SOVPC); 1-stearoyl-2-(9′-oxo-nonanoyl)-sn-glycero-3-phosphatidylcholine (SONPC); 1-stearoyl-2-glutaryl-sn-glycero-3-phosphochatidylline (SGPC); 1-stearoyl-2-azelaoyl-sn-glycero-3-phosphatidylcholine (SAzPC)) were used for external calibration quantification, as described earlier (Ademowo et al., 2020). All oxidized lipid standards were purchased from Avanti Polar Lipids (Alabama, United States) and Cayman Chemicals (MI, United States).

Organic solvents used, such as chloroform (CHCl_3_), methanol (MeOH), isopropanol, hexane (Hex), butyl acetate, and formic acid, were of HPLC grade. The toluene (Sigma 244511) and ethyl acetate (VWR 23880.290, EtAc) used were of the highest purity available (p.a.). Reagents, such as phosphoric acid (Sigma 1.00565) and copper(II) sulfate pentahydrate (Sigma C8027), were of the highest purity available. Sulfuric acid 95% (RP Normapur 20700) was purchased from Fisher Scientific. Butylated hydroxytoluene (BHT) was purchased from Merck (Dorset, United Kingdom).

### 2.2 Human umbilical vein cell culture conditions in normo- and hyperglycemia

Human umbilical cord vein endothelial cells (HUVECs) were harvested from three healthy donors. Recruited donors were normoglycemic (HbA1c < 42 mmol/mol) and confirmed to be free of HIV and hepatitis B. Participants provided informed consent, and the procedures were approved by the Ethics Board of the University of Münster (2009–537-f-S) in compliance with the Declaration of Helsinki principles. HUVECs were cultured and passaged in 1% gelatin-coated cell culture flasks (75 cm^2^) in Endothelial Cell Growth Medium (EGM™-2 BulletKit™, Lonza) supplemented with 5% fetal bovine serum (FBS) and 1% penicillin/streptomycin. The cells were placed in a humidified 5% CO_2_ cell culture incubator maintained at 37 °C. Once the cells reached 90% confluency, they were passaged into 1% gelatine-precoated 10-cm Petri dishes. The cells were passaged into 10-mm dishes until 70%–80% confluency in DMEM supplemented with 2% FBS and 1% P/S. As long-term exposure to high glucose (Glc) concentrations affects HUVEC membrane stiffness and cell integrity and function, leading to a lower number of live viable cells (Risso et al., 2001; Chen et al., 2013; Ciechanowska et al., 2021), the biochemical, biological, biomechanical, and functional responses reported in adaptation to extracellular glucotoxic environment are evident as early as 24 h of Glc exposure (20 mM) (Altannavch et al., 2004; Patel et al., 2013; Wang et al., 2024), suggesting that the plasma membrane could also be affected in this cross-talk. To investigate the lipid remodeling of the endothelial plasma membrane at the interface of blood and vessel wall, HUVECs were cultured under normal glucose (5 mM) or hyperglycemia conditions (20 mM glucose) containing EGM-2 media for 24 h.

### 2.3 Preparation of giant plasma membrane vesicles (GPMVs) from primary HUVECs

Plasma membrane was isolated as giant plasma membrane vesicles formed by chemical-induced vesiculation as previously described (Sezgin et al., 2012) with minor modifications. In brief, approximately 20 × 10^6^ cells were treated with [Ca^2+^]-containing GPMV buffer (10 mM HEPES, 150 mM NaCl, and 2 mM CaCl_2_, pH 7.4). Vesiculation was induced by 2 mM N-ethyl maleimide (NEM) supplemented with 25 mM paraformaldehyde (PFA) and left at 4 °C for 24 h. The GPMV release was checked under a microscope (Leica DMi1 inverted microscope). GPMV-rich supernatants were collected and centrifuged at 200 × *g* for 10 min to remove cell debris using an Eppendorf centrifuge and again in a Beckman ultracentrifuge at 20,000 × *g* for 1.5 h at 4 °C to pellet GPMV membranes for biochemical and lipidomic analysis. The prepared endothelial GPMVs were shipped in dry ice and stored in −80 °C upon arrival.

### 2.4 Analysis of GPMVs by Western blotting

Isolated HUVEC GPMVs were lysed in RIPA buffer (Thermo Fisher Scientific, United Kingdom), and protein content was quantified using the Pierce BCA Protein Assay Kit (Thermo Fisher Scientific) according to the manufacturer’s instructions. The protein fraction (10 µg) was resolved using SDS-PAGE, then transferred to a nitrocellulose membrane (Amersham, Sigma-Aldrich, Gillingham, United Kingdom) and incubated with Na^+^/K^+^ ATPase α Antibody (1:1,000, Cell Signaling Technology, Cat. #3010S), followed by fluorescence-conjugated secondary antibodies (IRDye® 800CW, anti-rabbit, #926–32211) from LI-COR Biosciences (Cambridge, United Kingdom). Bands were identified against prestained markers of known molecular weights (Thermo Scientific PageRuler Prestained Protein Ladder). Images were analyzed using ImageJ software.

### 2.5 Comprehensive analysis of GPMV lipid extracts by shotgun lipidomics

Lipid extracts were prepared from a volume of GPMVs corresponding to 100 µg protein and subjected to solvent extraction according to the protocol by Bligh and Dyer (1959). For quantitative lipidomics, internal standards were added prior to lipid extraction. The analysis of lipids was performed by direct flow injection analysis (FIA) using a triple quadrupole mass spectrometer (FIA-MS/MS) and a high-resolution hybrid quadrupole-Orbitrap mass spectrometer (FIA-FTMS), as described elsewhere (Höring et al., 2019; Höring et al., 2021). FIA-MS/MS was performed in positive ion mode using the analytical setup and strategy described previously (Liebisch et al., 2004). A fragment ion of *m/z* 184 was used for lysophosphatidylcholines (LPC) (Liebisch et al., 2002). The following neutral losses (NL) were applied: phosphatidylethanolamine (PE) and lysophosphatidylethanolamine (LPE) NL 141, phosphatidylserine (PS) NL 185, phosphatidylglycerol (PG) NL 189, and phosphatidylinositol (PI) NL 277 (Matyash et al., 2008). Sphingosine-based ceramides (Cer) and hexosylceramides (HexCer) were analyzed using a fragment ion of *m/z* 264 (Liebisch et al., 1999). PE-based plasmalogens (PE P) were analyzed according to the principles described elsewhere (Zemski Berry and Murphy, 2004). Cardiolipin was monitored by diglycerol fragment ions (Scherer et al., 2010). Annotation of glycerophospholipid species assumed even-numbered carbon chains only. A detailed description of the FIA-FTMS method was published recently (Höring et al., 2021). Triglycerides (TG), diglycerides (DG), and cholesterol esters (CE) were recorded in positive ion mode *m/z* 500–1,000 as [M + NH_4_]^+^ at a target resolution of 140,000 (at 200 m*/z*). CE species were corrected for their species-specific response (Höring et al., 2019). Phosphatidylcholines (PC), PC ether (PC O), and sphingomyelins (SM) were analyzed in negative ion mode *m/z* 520–960 as [M + HCOO]^−^ at the same resolution setting. Analysis of free cholesterol (FC) was performed by multiplexed acquisition (MSX) of the [M + NH_4_]^+^ of FC and the deuterated internal standard (FC[D7]) (Höring et al., 2019). Free fatty acids (FAs) were analyzed in negative ion mode *m/z* 150–450 as [M-H]^−^ dissolved in methanol/chloroform (5/1, v/v) containing 0.005% dimethylamine.

### 2.6 Screening of oxidized lipids in GPMVs by targeted liquid chromatography-multiple reaction monitoring mass spectrometry (LC-MRM-MS) strategies

Oxysterols, including 24-hydroxycholesterol (24-OHC), 25-hydroxycholesterol (25-OHC), 26-hydroxycholesterol (26-OHC), 7ß-hydroxycholesterol (7ß-OHC), and 7-ketocholesterol (7-KC), were quantified in GPMV lipid extracts by targeted liquid chromatography-multiple reaction monitoring (LC-MRM-MS) approaches, as described previously (Dias et al., 2018). Oxidized phosphatidylcholines (oxPC) were quantified by a targeted LC-MRM-MS approach in a QTrap mass spectrometer, as described earlier (Ademowo et al., 2020).

### 2.7 HPTLC analysis of GPMV extracts

HPTLC analysis was carried out in extracts of GPMVs prepared by extraction with tert-methyl butyl ether (Matyash et al., 2008) with slight modifications. In brief, 100 µL of deionized water was added to all samples and vortexed, and the samples were then transferred to glass tubes on ice. To each sample, 600 µL MeOH, 2 mL of MTBE, and 380 µL of water were added. All organic solvents contained 50 μg/mL BHT added on the day of extraction. Samples were automatically vortexed for 30 min at 4 °C–6 °C and centrifuged at 2000 × *g* for 5 min at 20 °C. The upper phase was collected into new 6-mL glass tubes. Then, 0.8 mL of MTBE:MeOH:H_2_O (10:3:2, v:v:v) was added to the remaining lower phase, vortexed, and centrifuged. Upper phases were collected. Samples were first partly dried under N_2_, following vacuum drying. Dried samples were kept under an inert atmosphere (argon) at −20 °C. On the analysis day, samples and standards were dissolved in MeOH:CHCl_3_ (2:1, v:v). Standard mixes contained equal masses of each lipid and were prepared as 1 μg/μL. HPTLC analysis was carried out as previously described (Dias Araújo et al., 2025) with the following modifications: the ATS4 dosage speed was 100 nL/s; 23 µL and 3 µL of sample and standard mix, respectively, were applied per band; the first band coordinates were X = 30 nm and Y = 8.0 mm; band length, thickness and distance were 8.0 mm, 1.0 mm, and 12.0 mm, respectively.

Imaging of HPTLC plates was carried out with a Fusion FX7 instrument (Vilber Loumat™), using epi white and blue lights (with a filter at λ = 590 nm). Images were treated with the image-processing software Fiji. Chromatograms were obtained after background removal (rolling ball radius 70 px; light background; smoothing disabled) and plotted using GraphPad Prism (v10).

### 2.8 Data and statistical analysis

Statistical analysis was done using univariate and multivariate methods. Statistics testing between groups for each lipid subclass was carried out using a standard two-sided, unpaired t-test assuming unequal variances with Bonferroni–Dunn correction for multiple comparisons in GraphPad Prism (version 8.4.3, San Diego, CA, United States). Statistical analysis of the shotgun lipid dataset was performed in MetaboAnalyst 6.0 software (Pang et al., 2024). Further statistical significance for the individual lipids in GPMV composition was assessed using multivariate analysis of variance (MANOVA) applied to the principal component analysis (PCA) scores calculated using normalized lipid concentrations. Prior to the analysis, the dataset was standardized sample-wise and variable-wise (mean-centered and divided by the standard deviation). First, the classification model was calculated by partial least squares-discriminant analysis with leave-one-out validation, and variables important in prediction (VIP) parameters for the variables were calculated. The 33 variables with the highest VIP values, that is, the highest contribution to the classification model, have been retained (shown in Supplementary Table S2). PCA was applied to the reduced dataset with the selected 33 variables. A MANOVA model used one factor, normo/hyperglycemic condition, and two dependent variables, PC1 and PC2 scores, and its statistical significance was tested. All algorithms were implemented in MATLAB® R2024b (MathWorks, Inc., Natick, MA, United States).

## 3 Results

### 3.1 Enrichment of plasma membrane fraction from human umbilical vein endothelial cells (HUVECs)

**FIGURE 1:**
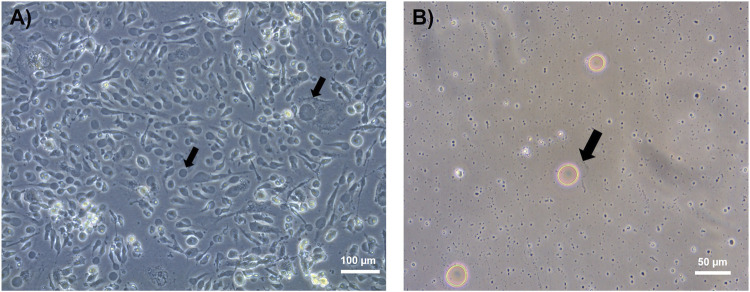
Light microscopy images of endothelial giant plasma membrane vesicles (GPMVs). Cell-derived GPMVs (marked with black arrows) formed by chemical-induced vesiculation are attached to endothelial cells prior to centrifugation [**(A)**, ×10 magnification)] and after the centrifugation step [**(B)**, ×20 magnification)].

**FIGURE 2:**
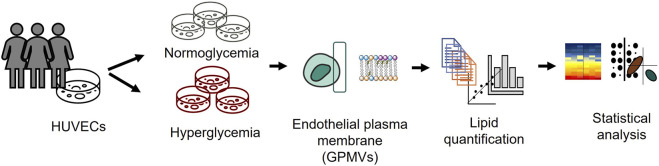
Experimental workflow for the characterization and quantification of (oxy)lipidome from endothelial GPMVs isolated from human donors.

Primary HUVECs harvested from recruited donors (3) were cultured under normo- (5 mM glucose) and hyperglycemic conditions (20 mM glucose). Glucotoxic conditions similar to those used in this study (20 mM of glucose for 24 h) have been described to promote the release of pro-inflammatory cytokine IL-6 and of adhesion molecules (ICAM-1, VCAM-1 and E-selectin) (Altannavch et al., 2004), induce a pro-oxidative environment by increased ROS production (Patel et al., 2013), inhibit cell proliferation, and increase barrier permeability (Zhao et al., 2015); traits associated with hyperglycemia-induced endothelial dysfunction (Fiorello et al., 2020; Tabit et al., 2010). HUVEC PM was isolated as giant plasma membrane vesicles (GPMVs) by chemical-induced vesiculation from normo- (Figure 1A) and hyperglycemic *in vitro* culture conditions. This gentle chemical method preserves lipid integrity, making GPMVs ideal for accurate, high-purity lipidomic profiling of endothelial cell membranes and an alternative to laborious and time-consuming density gradient ultracentrifugation, “peeling-off,” or commercial kit protocols in the enrichment of plasma cell membrane fractions. GPMVs were centrifuged for the removal of cell debris (Figure 1B) and used for lipidomic analysis. Enrichment of fractions in endothelial GPMVs was confirmed by the presence of the band corresponding to Na^+^/K^+^ ATPase (Supplementary Figure S1), a typical PM protein (Lingrel and Kuntzweiler, 1994). HUVEC-derived GPMVs were analyzed for their lipid composition and content. An overview of the experimental design used in this work is depicted in Figure 2.

### 3.2 Screening of GPMV lipid compositions in normoglycemia HUVECs

Analysis of endothelial GPMV lipid extracts by shotgun lipidomic mass spectrometry (MS)-based approaches (direct infusion) resulted in the annotation of 251 lipid species from 18 lipid classes (Supplementary Table S1) along with 13 oxidized lipids (oxysterols and oxidized phosphatidylcholines) identified by targeted multi-reaction monitoring LC-MS approaches. Lipids identified were named according to the LIPID MAPS nomenclature guidelines (Liebisch et al., 2019). All species in PC, PC-O, SM, PE, PS, PI, PG, CL, DG, and TG, except for Cer, HexCer, and PE-P, were annotated according to the total number of carbon atoms:double bonds (e.g., PE 36:2; PI 38:4; CL 34:2_36:3; TG 54:6). Among the 32 PE-P lipids composing the HUVEC PM lipidome, 13 were not found on the LIPID MAPS database (noted as N/A in Supplementary Table S1). Nonetheless, because of the analysis strategy adopted for lipid characterization (direct infusion of lipid extracts into the MS source without prior chromatographic separation), the number of molecular lipids composing the endothelial PM lipidome is expected to be greater than the 251 lipids annotated in this study, as the possible contribution of structural and positional isomers cannot be discarded. For example, screening PE lipids by neutral loss scanning (NLS) of the signature fragment with 141 a.m.u. in the (+)ve ion mode shows the predominance of the diacyl-phosphatidylethanolamine PE 36:2 in endothelial GPMVs (Supplementary Material, Supplementary Figure S2). The PE 36:2 lipid can be attributed to the presence of PE 18:0_18:2 or PE 18:1_18:1 or even to the cumulative contribution of both structures, which were previously identified in human pulmonary artery EC (Hirata et al., 2021) and in vesicles from epithelial-like cells (Llorente et al., 2013). Similarly, ether-linked phosphatidylcholines (PC-O) with alkyl-acyl structure can also be attributed to their alkenyl-acyl-phosphatidylcholines plasmalogen isomers, which can only be distinguished using (chromatographic) separation and tandem MS (Koch et al., 2020). Likewise, screening using precursor ion screening (PIS) of the fragment at *m/z* 264 detects Cer and HexCer with a sphingoid base with two hydroxyl groups. Other complex sphingolipids and phosphorylated inositol lipids, typically enriched in PM fractions and vesicles (Gerl et al., 2012; Llorente et al., 2013), were not screened due to poor extraction performance by two-phase solvent systems (Matyash et al., 2008; Reis et al., 2013).

### 3.3 Cholesterol is the predominant lipid composing GPMVs in the HUVEC membrane

Absolute amounts of quantified lipids (nmol) normalized against the protein content in each GPMV fraction (nmol/mg protein), as shown in Supplementary Table S1, are summarized in Table 1. As the amount of total lipids in GPMV extracts (normalized to nmol/mg protein) is different (Table 1), the content of lipids in GMPV extracts was converted to percentage values (mol%) for comparison purposes. This conversion accounted for any analytical variance in the extraction efficiency between samples.

**TABLE 1:** Lipid class composition (mol %) of endothelial GPMVs isolated from HUVECs collected from three donors and grown under normo- and hyperglycemia conditions. GPMV lipid extracts quantified by targeted and untargeted shotgun mass spectrometry (MS) approaches normalized to protein content (nmol/mg protein) can be found in Supplementary Table S1. Significance was assessed using a t-test corrected using the Bonferroni–Dunn method (GraphPad software).

Method (mass analyzer/ionization mode)	Lipid class	Normoglycemia			Hyperglycemia			p-value
		Donor #1	Donor #2	Donor #3	Donor #1	Donor #2	Donor #3	
*Phospholipids*								
QQQ+ SRM Q3mz184	LPC	0.548	0.424	0.423	0.443	0.425	0.376	0.3360
FTMS− 520–960 mz	PC	19.932	19.024	17.070	19.353	18.430	16.782	0.6889
FTMS− 520–960 mz	PC-O	1.145	1.116	0.715	1.224	1.212	0.625	0.9123
QQQ+ SRM NL141	LPE	1.405	0.960	1.453	1.123	0.886	1.067	0.2249
QQQ+ SRM NL141	PE	6.313	7.125	6.697	5.752	6.665	7.215	0.7479
QQQ+ SRM	PE-P	2.115	2.368	2.394	2.479	2.569	2.238	0.3632
QQQ+ SRM NL185	PS	4.769	5.567	6.015	5.747	5.628	5.854	0.4734
QQQ+ SRM NL277	PI	5.625	5.615	4.442	4.886	6.169	4.232	0.8582
QQQ+ SRM NL189	PG	0.054	0.082	0.364	0.073	0.094	0.220	0.7473
QQQ+ SRM DG-fragm	CL	0.084	0.114	0.155	0.086	0.141	0.122	0.9618
*(Glyco)sphingolipids*								
FTMS− 520–960 mz	SM	7.058	7.394	7.221	7.497	6.857	7.610	0.7217
QQQ+ SRM Q3mz264	Cer	0.176	0.196	0.214	0.185	0.206	0.188	0.8641
QQQ+ SRM Q3mz264	HexCer	0.031	0.024	0.018	0.028	0.024	0.020	0.9434
*Storage lipids*								
FTMS+ 500–1,000 mz	DG	1.732	1.542	1.686	1.837	1.714	1.511	0.7745
FTMS+ 500–1,000 mz	TG	0.702	0.795	1.360	0.731	1.263	0.643	0.8079
FTMS+ 500–1,000 mz	CE	0.180	0.275	0.308	0.188	0.360	0.198	0.9373
FTMS− 150–450 mz MA	FA	3.325	3.099	4.061	2.049	3.726	4.366	0.8858
*Free Sterols*								
FTMS+ MSX	FC	44.81	44.28	45.40	46.32	43.63	46.73	0.5155
	Total lipids (nmol/mg protein)	637.88	717.86	581.09	537.54	643.98	525.78	0.2341
	Chol/PL ratio	1.12	1.08	1.20	1.17	1.07	1.25	0.6582
	PE/PS ratio	1.77	1.71	1.51	1.43	1.64	1.61	0.3698
Oxidized lipids								
QTrap+ MRM	Total oxysterols (pmol/mg protein)	902.3	1311.6	1091.3	1680.8	1027.6	576.5	0.9018
QTrap+ MRM *m/z* 184	Total oxPC (pmol/mg protein)	22.2	26.8	18.4	16.8	19.3	9.6	0.0182
	Protein (mg/mL)	0.69257	1.07436	0.79554	0.46168	1.38647	1.03171	0.7359

**FIGURE 3:**
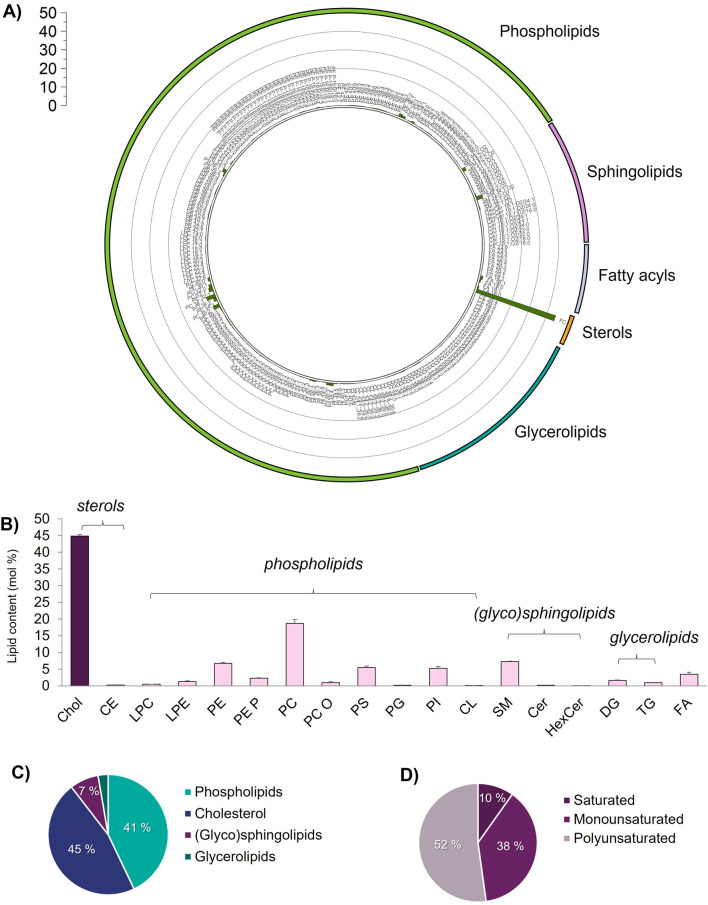
Global lipid characterization of giant plasma membrane vesicles (GPMVs) isolated from primary human endothelial cells grown under normoglycemic conditions, quantified by shotgun lipidomics in endothelial GPMVs. **(A)** Individual lipid content (mol%); scale bar depicts mol (%), data is available in Supplementary Table S1; **(B)** lipid class content (mol%), **(C)** lipid category content (mol%), and **(D)** phospholipid acyl chains saturation content (mol%). Panel of lipid classes identified include Chol, cholesterol; CE, cholesteryl esters; LPC, lysophosphatidylcholine; LPE, lysophosphatidylethanolamine; PE, phosphatidylethanolamine; PE_P, plasmenyl phosphatidylethanolamine; PC, phosphatidylcholine; PC_O, ether-linked phosphatidylcholine; PS, phosphatidylserine; PG, phosphatidylglycerol; PI, phosphatidylinositol; CL, cardiolipin; SM, sphingomyelin; Cer, ceramides; HexCer, hexosylceramides; DG, diacylglycerides; TG, triacylglycerides; FA, fatty acids. The total sum of percentages in C) makes 94% as fatty acyls (FA) and cholesteryl esters (CE) were not included. Error bars depict standard error (SD, *n* = 3).

The individual lipid content (Figure 3A) data show that cholesterol is the most abundant lipid in the HUVEC PM lipidome, reaching 45 mol%, and is higher than other lipid subclasses (Figure 3B). In addition to cholesterol, our large-scale lipidomic analysis shows that endothelial GPMVs are largely composed of phospholipids (41 mol%), as shown in Figure 3C, with the predominance of PC (19 mol%), PE (9 mol%), PS (5 mol%), and PI (5 mol%), together with small amounts of (glyco) sphingolipids (GSL, 7 mol%) and storage glycerolipids (2 mol%), shown in Figure 3B. The ratio of Chol:PL:(G) SL of 45:41:7 (mol%) found in GPMV fractions of primary HUVECs is in agreement with the values found for the apical leaflet fraction isolated from MDCK (Gerl et al., 2012) and other vesicle preparations (exosomes) from a prostate cancer cell line (Llorente et al., 2013). The most abundant phospholipids were PC and aminophospholipids, PE, and PS, consistent with the phospholipidome reported in the GPMVs of various epithelial-like cell lines (Symons et al., 2021). The phospholipid fraction is mostly composed of polyunsaturated acyl chains (52%, ≥2 double bonds) with monounsaturated lipids accounting for 38 mol% and saturated ones accounting for less than 10 mol% (Figure 3D). Monounsaturated phospho- and sphingolipids, PC 32:1, PC 34:1, and SM 34:1, are most likely localized in the outer leaflet, whereas monounsaturated PE 36:1, PS 36:1, PE 36:2, PI 38:4, and other polyunsaturated PL are likely confined to the inner leaflet of the endothelial bilayer (Pabst and Keller, 2024; Schütz and Pabst, 2023). (Glyco) sphingolipids, including SM, Cer, and HexCer, accounted for only 7 mol% of the total quantified lipids (Figure 3C). While this value is similar to those reported in the apical leaflet of Madin–Darby kidney cells (Gerl et al., 2012) and in the PM of epithelial mammalian cell lines (Symons et al., 2021), the contribution of GSL to the total endothelial PM lipidome is likely higher, as complex GSLs (e.g., lactosylceramides, sulfatides, gangliosides and globosides, and Forssman glycolipids), already reported in HUVECs (Müthing et al., 1999), were not included in the shotgun workflow. Storage lipids (DG and TG) and FA levels are low (Figure 3B), and in agreement with those reported previously in mammalian GPMV fractions (Symons et al., 2021).

**FIGURE 4:**
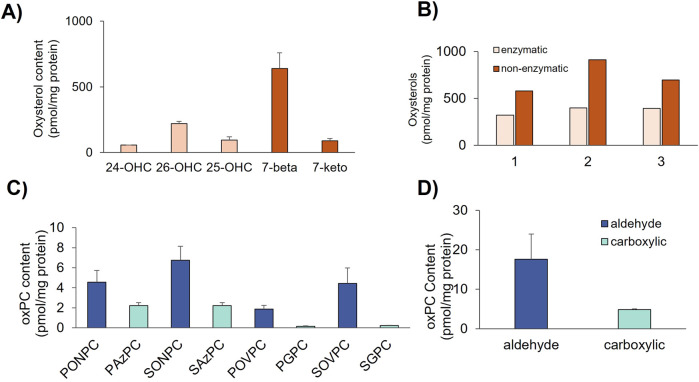
Endothelial GPMV oxylipidome in normoglycemia. Bar graphs represent **(A)** profile and content (pmol/mg protein) of oxysterols formed by enzymatic (24-OHC, 25-OHC, and 26-OHC, light orange) and non-enzymatic pathways (7-betaOHC and 7-ketoC, dark orange); **(B)** total amount (pmol/mg protein) of enzymatic oxysterols (light orange) and non-enzymatic oxysterols (dark orange) in individual donors; **(C)** profile and content (pmol/mg protein) of short-chain (truncated) oxidized phosphatidylcholines (oxPC) bearing a palmitoyl-linked acyl chain (PONPC, PAzPC, POVPC, and PGPC) and a stearoyl-linked (SONPC, SAzPC, SOVPC, and SGPC) acyl chains; and **(D)** total content of oxidized short-chain (truncated) oxPC bearing a terminal aldehyde (dark blue) and carboxylic moiety (light blue). Error bars depict standard error (SD, *n* = 3).

Given the predominance of cholesterol (45 mol%) and PC lipids (∼20 mol%) in the HUVEC PM lipidome, oxidized products of cholesterol (oxysterols) and phosphatidylcholines (oxPC) were also analyzed. Oxysterols screened included those formed by enzymatic modification, such as 24-, 26-, and 25-hydroxycholesterol, as well as those formed by non-enzymatic free radical modification (e.g., hydroxyl, superoxide), such as 7-β-hydroxycholesterol and 7-keto-cholesterol. The amount of total oxysterols in normoglycemic GPMVs ranged from the nanomolar (625 nM, 0.902 nmol/mg protein) to the submicromolar range (1,409 nM, 1.311 nmol/mg protein). Among oxysterols, the predominance of 7-β-hydroxycholesterol (Figure 4A) was responsible for the predominance of non-enzymatic oxysterols in all three donors (Figure 4B). Analysis of truncated oxidized phosphatidylcholines (oxPC, Figure 4C) shows that oxPCs bearing a terminal aldehyde moiety linked to palmitoyl- and stearoyl-linked acyl chains (PONPC, SONPC, and SOVPC) predominate in GPMV extracts (Figure 4D) over those with a terminal carboxylic group (PAzPC, SAzPC, PGPC, and SGPC).

### 3.4 HUVEC plasma membrane undergoes lipid remodeling under glucotoxic conditions

**FIGURE 5:**
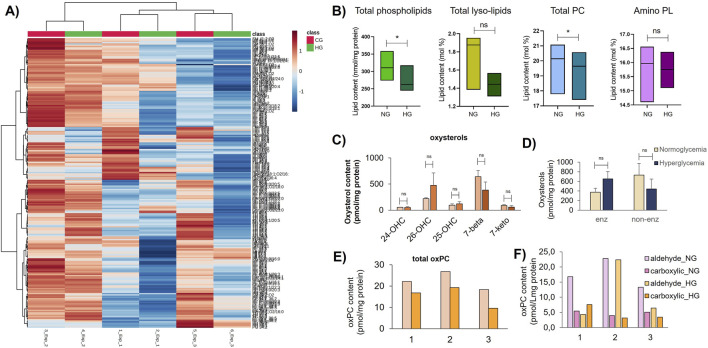
Effect of hyperglycemia on endothelial GPMV (oxy)lipidome. **(A)** Hierarchical clustering heatmap of log_2_ converted values for individual lipid species detected in the normoglycemic group (CG, red) and the hyperglycemic group (HG, green) across three donors, showing similarities and disparities in lipid concentrations (generated in MetaboAnalyst). Color code (right-hand side) refers to lipid concentration (log-transformed values); **(B)** lipid subclass content, concerning total phospholipids (pmol/mg protein), total lysolipids (mol%, LPC + LPE), total phosphatidylcholines (mol%, diacyl- and plasmenyl-PC), and aminophospholipids (PE + PS, mol%). Lighter colors in boxplots represent normoglycemia and darker colors correspond to hyperglycemia; **(C)** concentration (pmol/mg protein) of enzymatic and non-enzymatic oxysterols screened in normo- (lighter colors) and hyperglycemia (darker colors); **(D)** total oxysterol content (pmol/mg protein) in normo- and hyperglycemia; **(E)** total content (pmol/mg protein) of oxidized short-chain (truncated) phosphatidylcholines (oxPC) in GPMVs under normo- (pink) and hyperglycemia (orange) in individual donors (*n* = 3); and **(F)** content of truncated oxPC with aldehyde and carboxylic terminal groups in acyl chains in GPMVs under normo- and hyperglycemic conditions in individual donors. Lines in boxplot graphs depict the medians of min and max values. Error bars depict standard error (SD), *n* = 3. Significance was determined at *p* < 0.05 (*); ns, not significant.

Exposure of primary HUVECs to glucotoxic conditions shows a decreasing trend in the amount of total lipids compared to normoglycemia (Supplementary Table S1) that is reflected across the GPMV lipidome in the different donors (Figure 5A). Changes to the GPMV lipidome occur in total phospholipids and specifically to total phosphatidylcholines, whereas total lysolipids (lysophosphatidylcholines (LPC) and lyso-ethanolamines (LPE)), aminophospholipids (Figure 5B), and polyunsaturated SM (Supplementary Figure S3A) show a decreasing trend. In consequence, the decrease in total content of phospholipids in hyperglycemia results in a slight overall increase in the cholesterol-to-phospholipid ratio (Chol/PL). The increase in the Chol/PL ratio in adaptation to hyperglycemia has major implications in membrane organization and dynamics, resulting in increased membrane rigidity as previously reported in RBCs and HUVECs collected from patients with type 2 diabetes mellitus (T2DM) (Allen et al., 2006; Wang et al., 2024). Increased membrane rigidity has a clear impact on the diffusion of metabolites and gases (Miersch et al., 2008; Subczynski et al., 2017; Subczynski et al., 1996). In addition to changes in the Chol/PL ratio, several individual molecular species of lysolipids, phospholipids, and sphingolipids are altered in adaptation to hyperglycemia (Supplementary Table S1). The analysis of PM fractions from individual donors, rather than pooled PM fractions, highlights the interindividual variability between donors (Supplementary Figure S3A) as well as changes to lipid content, which, while small, are consistent across all three donors (bar graphs in Supplementary Figure S3A). Multivariate analysis conducted on the lipidomic shotgun datasets by partial least square discriminant analysis (PLS-DA) revealed a panel of 33 molecular lipids contributing to the discrimination between normo- and hyperglycemia, as shown by the variable importance in the projection (VIP) index (Supplementary Figure S3B), where most of the discriminative individual lipids are species of PC and PE subclasses. Principal component analysis (PCA) applied to the 33 highest-ranking variables (Supplementary Table S2) revealed clustering of samples according to glycemia as shown by the score plot (Supplementary Figure S3C), where the first two PCs accounted for 86% of the total variance.

HPTLC analysis conducted on the GPMV extracts to tentatively complement the information on the GSL lipids revealed faint bands in the polar lipids elution zone corresponding to the potential presence of complex GSL (bands marked with * and **, Supplementary Figure S4). In addition, due to the low abundance of glycerolipids in PM extracts, the band was assigned to Cer, which showed a marked decrease in hyperglycemia in donors studied (Supplementary Figure S4). This result differs from the mass spectrometry-based findings (Table 1), hinting that, in addition to the seven sphingosine Cer structures (Cer d18:1) screened (Supplementary Table S1), endothelial PM may contain other dihydroceramides (dhCer, hydroxy-sphinganine) structures not screened through the shotgun approach. Differences observed between MS-based approaches (Bligh and Dyer protocol) and the HPTLC approach (Matyash protocol) may also be partially attributed to the distinct extractability of SL in organic biphasic solvent mixtures (Matyash et al., 2008; Reis et al., 2013). In view of the results here obtained, HPTLC holds great potential for full screening of biological samples prior to the expensive and highly skilled (LC)MS-based approaches (Dias Araújo et al., 2025).

In addition, exposure of HUVECs to glucotoxic conditions resulted in an increase in 26-hydroxycholesterol and a decrease in 7-beta-hydrocholesterol (Figure 5C) with the predominance of enzymatic oxysterols over non-enzymatic oxysterols (Figure 5D). Results hint at the upregulation of enzymes CH25H and CYP27A1 in hyperglycemia. Remarkably, hyperglycemia led to a decreasing trend in total oxPC levels in the GPMVs of all donors (Figure 5E), mostly due to the decreased content of oxPC with a terminal aldehyde moiety (Figure 5F).

## 4 Discussion

For many years, the endothelial barrier was regarded as inert. It is now widely accepted that the endothelium plays a key role in the blood–endothelial cross-talk and is actively involved in mediating the inflammatory response, ultimately contributing to endothelial homeostasis and vascular health (Campinho et al., 2020; Hsieh et al., 2014). Being at the interface of blood and blood vessels, the endothelial plasma membrane (PM) is the first point of contact with external stimuli in circulation (lipoproteins, cells, and metabolites). Acting as the support of enzymes and receptors responsible for signaling and trafficking events, improved knowledge of the lipid environment in endothelial PM is of the utmost importance to expand our understanding of the barrier properties and the functional changes taking place in adaptation to hyperglycemia.

Through the quantification of 264 lipid species from 20 subclasses, including cholesterol and oxidized lipids, our study provides the first comprehensive characterization of PM lipidome in primary HUVECs. To the best of our knowledge, this is the first study describing the lipidome in chemically induced PM vesicles from primary human cells, as studies published in the literature are limited to immortalized cell lines (Alter et al., 2023; Azbazdar et al., 2023; Kheradmandi et al., 2023; Symons et al., 2021) known to contain a lipid composition distinct from that of their primary cells (Reis et al., 2024; Symons et al., 2021). A second innovative aspect of our study is the inclusion of cholesterol in our lipidomic strategy, as studies published in the literature focus on the phospholipidome, completely overlooking the contribution of cholesterol to the PM lipidome (Alter et al., 2023; Azbazdar et al., 2023; Kheradmandi et al., 2023; Symons et al., 2021). Our study shows that cholesterol comprises nearly half (45 mol%) of the lipid composition in endothelial PM, a value that is well in line with that reported in the PM of primary human pulmonary aortic endothelial cells by imaging approaches (Buwaneka et al., 2021). Interestingly, such a high amount of Chol found here is in close agreement with that described in the apical leaflet in canine kidney cells (Gerl et al., 2012) and extracellular vesicles (EVs) from epithelial prostate cancer PC-3 cell line (Llorente et al., 2013). While EVs, also termed exosomes, which are secreted by most cells under physiological conditions and released to the extracellular environment, are inherently distinct from GPMVs, which are formed *in vitro* by the addition of chemical stressors, they are both vesicle particles formed during budding/blebbing events involving the PM. As such, they retain much of the PM lipid and protein complexity of living cells, thus serving as good models of biological membranes (Llorente et al., 2013; Sezgin et al., 2012; Skotland et al., 2023). Remarkably, both cell-derived GPMVs and EVs possess a unique lipid composition enriched in cholesterol, phospholipids, and sphingolipids (Llorente et al., 2013; Sarabipour et al., 2015), which are crucial to maintain the barrier properties in cells. While the majority of Chol is exclusively confined to the apical (exoplasmic) leaflet (Buwaneka et al., 2021; Pabst and Keller, 2024), Buwaneka and colleagues found that <1 mol% of membrane Chol was located at the inner leaflet (Buwaneka et al., 2021). This uneven distribution of Chol across the PM bilayer is crucial for the outer leaflet to be sufficiently impermeable to the external environment and for the inner leaflet to be sufficiently fluid, thus providing the optimum lipid environment for the structural and functional modulation of integral membrane proteins involved in key cellular and signaling events (Deliconstantinos et al., 1995; Harayama and Antonny, 2023; Lorent et al., 2020; Schütz and Pabst, 2023; Tiberti et al., 2020). The amount of Chol found in our study is higher than that currently used in experimental and computational studies that range from 18 mol% to 40 mol% (De Almeida et al., 2003; Ingólfsson et al., 2014; Kaiser et al., 2009; Lorent et al., 2020; Wilson et al., 2021), reinforcing the need to upgrade membrane models when investigating membrane organization. The presence of such high amounts of Chol in endothelial PM will surely advance the current knowledge on cholesterol-mediated cell signaling in diet- and age-related chronic diseases. For example, recent investigations found that membrane Chol tightly regulates the low-density lipoprotein receptor (LDLR) (Morales et al., 2023). However, the conformation of the LDLR-membrane immersed moiety in the cholesterol-rich endothelial bilayers remains elusive.

Expanding our shotgun analysis to include the lipid remodeling in adaptation to hyperglycemia introduces a second innovative aspect. Our data revealed that under *in vitro* hyperglycemia conditions, HUVECs adapt the PM lipid composition showing a consistent trend with: 1) decreased lipid content; decreased phospholipid content with a concomitantly increased Chol/PL ratio (Table 1), 2) decreased trend of polyunsaturated LPC, LPE, PC, and PE species and of monounsaturated SM and increase in PS (Supplementary Figure S3A), 3) a marked decrease in Cer levels (Supplementary Figure S4), 4) a shift toward the predominance of enzymatic (tail-oxidized) oxysterols (Figure 5C), and 5) a decrease in truncated oxPC content (Figure 5E). Although trends observed were consistent across all donors, the interindividual variability allied with the low sample number (*n* = 3) contributed to the poor statistical significance.

Because GPMVs do not retain the membrane’s lipid asymmetry, the changes outlined are associated with changes to both the outer and inner leaflet compositions. Changes noted are likely to alter the lipid environment in which membrane proteins (surface receptors and enzymes) are embedded and ultimately affect the activation of downstream signaling events involved in the proper endothelial function events (Hsieh et al., 2014; Moessinger et al., 2020). For instance, at physiological pH, PE lipids together with other anionic phospholipids such as PI, PS, and PG appear to contribute to the net negative charge of the membrane (Ribeiro et al., 2012) and are active participants in the modulation of glucose transporter (GLUT) activity (Hresko et al., 2016). Likewise, the increased Chol/PL ratio observed in this study, associated with increased rigidity and membrane stiffening in hyperglycemia (Allen et al., 2006; Wang et al., 2024), appears to be one of the cell’s responses to mechanical and other environmental stimuli, including age and disease (Hashimoto et al., 1999; Yamamoto and Ando, 2013; Yamamoto and Anto, 2015). The increase in the Chol/PL ratio has a clear impact on NO bioavailability, not only by modulating eNOS activity localized in cholesterol-rich domains in endothelial PM (Deliconstantinos et al., 1995; Zhang et al., 2006) but also by regulating NO diffusion reaching the vascular tissue (Miersch et al., 2008). In addition, the marked decrease in Cer levels (Supplementary Figure S4) may likely cause the membrane’s cholesterol–sphingolipid domains to be slightly more disordered, given the described effect of Cer in complex raft-like artificial membranes characterized with a coexisting liquid ordered/liquid disordered (*l*_*o*_/*l*_*d*_) phase (Castro et al., 2014), likely altering lipid–protein interactions and the cellular signaling events propagating the endothelial dysfunction (Senthilkumar et al., 2024).

Similarly, the shift toward the predominance of enzymatic oxysterols (Figure 5C) and decreased oxPC (Figure 5E) is likely to impact the membrane’s organization and biomechanical properties (Ayee et al., 2017; Byfield et al., 2006; Shentu et al., 2012). The predominance of enzymatic (tail-oxidized) oxysterols renders more rigid biomembranes, unlike their non-enzymatic (ring-oxidized) counterparts that were shown to increase the membrane permeability in model membranes (Kulig et al., 2015). Similarly, the decrease in oxPC observed in hyperglycemia for all individuals (Figure 5E) may lead to an increase in membrane rigidity, as previous findings by Ayee and colleagues (2017) found that the incorporation of approx. 2% of arachidonoyl-based oxPC to *in vitro* cultured endothelial cells resulted in the decrease of lipid order, consistent with a fluidizing effect on the membrane (Ayee et al., 2017). Although oxPC in endothelial PM accounted for only 0.02% of the total PC levels, this value is well below that considered so far in experimental and theoretical membrane model studies (Ayee et al., 2017; Wong-Ekkabut et al., 2007), prompting a revision of the composition of model membranes. In addition, the decreasing trend of oxPC with reactive aldehyde moiety observed in hyperglycemia (Figure 5F) hints at the occurrence of protein–lipid cross-linking reactions responsible for the unusual decrease of oxPC in stress conditions. Considering the changes noted to the lipid families and individual lipids (Figure 5B; Supplementary Figure S4), these are likely to alter the membrane’s lipid environment and likely expose amino groups in membrane proteins to become physically accessible to cross-linking modification by oxPC with terminal reactive aldehyde groups, leading to the formation of protein-oxPC adducts (Gao et al., 2020; Reis et al., 2006; Spickett et al., 2013). These protein–lipid cross-linking reactions, together with other lipid–lipid adducts formed between glucose and aminolipids PE and PS, and evidenced by the decrease in specific polyunsaturated PE lipids (Supplementary Table S1; Supplementary Figure S3A), change the PM organization and are likely contributors to the rigidity of endothelial membranes reported in age and T2DM (Chen et al., 2013; Hashimoto et al., 1999; Wang et al., 2024). Non-enzymatic cross-linking reactions are a well-known mechanism of protein *in vivo* modification in T2DM (Wautier and Schmidt, 2004), likely to affect lipid–protein interactions, endothelial membrane biophysics, and vascular permeability, contributing to the endothelial dysfunction and associated vascular complications typical of T2DM (Chen et al., 2013; Fiorello et al., 2020; Oak et al., 2003). While protein–lipid and lipid–lipid adducts were not screened, our result highlights the need for further studies to provide more accurate estimates on the (patho) physiologically relevant concentrations of oxidized lipids in cholesterol-rich membranes, glycated-PE adducts, and their spatial distribution within the lipid bilayer (Reis, 2017).

While our findings reflect the PM lipid remodeling of HUVECs in adaptation to glucose-stressed conditions, comparison of our findings to *in vivo* endothelial PM remodeling in hyperglycemia should be carefully evaluated, bearing in mind the strengths and limitations associated with the adopted experimental design. First, while the use of primary HUVECs harvested from T2DM donors would be more physiologically relevant, the use of primary HUVECs cultured *in vitro* under glucose-stressed conditions offers the advantage of investigating the effect of hyperglycemia without the associated confounding factors (age, diet, medication, others). Second, harvested primary HUVECs used in this and previous studies (Hirata et al., 2021; Hong et al., 2024) were cultured *in vitro* under static atmospheric conditions, not really mimicking the hemodynamic forces in circulation and the physiological normoxia conditions (*p*O_2_ 3–5 kPa, 22–40 mmHg) in the endothelium (Keeley and Mann, 2019). Third, while GPMVs provide an obvious approach to study biological membrane heterogeneities, they represent a state of thermodynamic equilibrium that is intimately related to the limitations of vesiculation agents and the cellular events taking place during blebbing and vesicle formation (Keller et al., 2009; Levental et al., 2011; Sezgin et al., 2012). Fourth, cell-derived GPMVs have routinely been used for lipidomic analysis (Azbazdar et al., 2023; Plochberger et al., 2020; Symons et al., 2021), although, like other membrane isolation methods such as commercial kits, ultracentrifugation, and “peeling-off” protocols (Bünger et al., 2009; Gerl et al., 2012; Li et al., 2019), some cross-contamination is expected (Sezgin, 2022). Fifth, our collaborative work highlights the challenges surrounding the implementation of standardized protocols across research laboratories. Despite major efforts undertaken by consortia to standardize and harmonize the lipidomics pipeline to minimize contamination and improve the extraction performance and lipidome coverage in clinical and cell lipidomics (Bowden et al., 2017), in practice, research groups are more likely to adopt procedures already implemented in their own laboratories. Finally, while shotgun MS-based approaches have been validated in the quantification of lipid species in biofluids, cultured cells, and tissues (Gallego et al., 2018), the methodology presents some limitations when compared to other LC-MS approaches that may render underestimation of lipid species with poor ionization efficiency.

Moreover, the heterogeneity of endothelium across the vascular tree (Aird, 2007; McAleese et al., 2025) and the cell-type specificity of the (phospho) lipidome (Murphy et al., 1992; Symons et al., 2021) prompts the need to expand this approach other EC phenotypes (e.g., heart, lung, and blood brain barrier) to broaden our knowledge on the lipid remodeling in EC harvested in other scenarios with associated endothelial dysfunction such as age, obesity, and hypertension with multimorbidities.

In conclusion, we describe the first exploratory characterization lipidome study of endothelial plasma membrane isolated from primary HUVECs cultured *in vitro* under normoglycemia conditions and the lipid remodeling in adaptation to hyperglycemia. Our shotgun approach reveals a snapshot of the myriad hyperglycemia-induced changes to the cholesterol-to-phospholipid ratio, to lipid subclasses located at the outer and inner leaflets, and the shift toward the predominance of enzymatic (tail-oxidized) oxysterols. These changes suggest a profound impact of hyperglycemia on the membrane’s biophysical and biomechanical properties, which could account for the endothelial dysfunction in hyperglycemia conditions, a hallmark of diabetes-related vascular complications.

In spite of its exploratory character, findings from this study, together with the already known asymmetric transbilayer distribution of cholesterol and phospholipid classes, provide the basis for future *in silico* investigations evaluating the role of membrane lipid environments on protein–lipid interactions, particularly those involved in sugar (GLUT) and lipid metabolism (LDL receptor), vascular function (eNOS), and in lipid–drug interactions governing complex signaling cascades in disease conditions.

## Data Availability

The original contributions presented in the study are included in the article/Supplementary Material; further inquiries can be directed to the corresponding authors.

## Glossary

a.m.u.: Atomic mass units
BHT: Butylated hydroxytoluene
Chol: Cholesterol
CH25H: Cholesterol 25-hydrolase
CYP27A1: Cytochrome P450 Family 27 Subfamily A Member 1
CYP 46A1: Cytochrome P450 Family 46 Subfamily A Member 1
EC: Endothelial cell
eNOS: Endothelial nitric oxide synthase
FBS: Fetal bovine serum
GLUT: Glucose transporter
GPMV: Giant plasma membrane vesicle
GSL: Glycosphingolipids
HPTLC: High-performance thin-layer chromatography
HUVECs: Human umbilical vein endothelial cells
LDL: Low-density lipoprotein
LC-MS: Liquid chromatography coupled to mass spectrometry detection
MeOH: Methanol
MTBE: Methyl-tert-butyl ether
NEM: N-Ethyl-maleimide
NO: Nitric oxide
PAzPC: 1-Palmitoyl-2-azelaoyl-sn-glycero-3-phosphatidylcholine
PG: Phosphatidylglycerol
PGPC: 1-palmitoyl-2-glutaroyl-sn-glycero-3-phosphatidylcholine
PC: Phosphatidylcholine
PE: Phosphatidylethanolamine
PCA: Principal component analysis
PI: Phosphatidylinositol
PL: Phospholipid
PM: Plasma membrane
PONPC: 1-Palmitoyl-2-(9-oxo-nonanoyl)-sn-glycero-3-phosphatidylcholine
POVPC: 1-Palmitoyl-2-(5-oxo-valeroyl)-sn-glycero-3-phosphatidylcholine
RBC: Red blood cells
SAPC: 1-Stearoyl-2-arachidonoyl-sn-glycero-3-phosphatidylcholine
SAzPC: 1-Stearoyl-2-azelaoyl-sn-glycero-3-phosphatidylcholine
SGPC: 1-Stearoyl-2-glutaroyl-sn-glycero-3-phosphatidylcholine
SLPC: 1-Stearoyl-2-lineloyl-sn-glycero-3-phosphatidylcholine
SONPC: 1-Stearoyl-2-(9-oxo-nonanoyl)-sn-glycero-3-phosphatidylcholine
SOVPC: 1-Stearoyl-2-(5-oxo-valeroyl)-sn-glycero-3-phosphatidylcholine
VIP: Variables important in prediction
T2DM: Type 2 diabetes mellitus
