# Correction: The endothelial plasma membrane lipidome and its remodeling under hyperglycemia: an exploratory study

**DOI:** 10.3389/fmolb.2026.1814292

**Published:** 2026-04-02

**Authors:** Ana Reis, Yahya Sohrabi, Lorena Diaz-Sanchez, Ana Rita Dias Araújo, Merle Leffers, Bruno Antonny, Alisa Rudnitskaya, Rui Vitorino, Irundika H. K. Dias

**Affiliations:** 1 REQUIMTE/LAQV, Departamento de Química e Bioquímica, Faculdade de Ciências, Universidade do Porto, Rua do Campo Alegre, Porto, Portugal; 2 Department of Cardiology I, Coronary, Peripheral Vascular Disease and Heart Failure, University Hospital Münster, University of Münster, Münster, Germany; 3 Aston Medical School, College of Health and Life Sciences, Aston University, Birmingham, United Kingdom; 4 Université Côte d’Azur, CNRS and Inserm, Institut de Pharmacologie Moléculaire et Cellulaire, Valbonne, France; 5 CESAM/Department of Chemistry, University of Aveiro, Aveiro, Portugal; 6 iBIMED, Institute of Biomedicine, Department of Medical Sciences, Aveiro, Portugal

**Keywords:** cholesterol, giant plasma membrane vesicles, human umbilical vein endothelial cells, oxidized phospholipids, oxysterols, shotgun mass spectrometry

Supplemental material Data Sheet 1 was omitted. The file has now been published.

The original article has been updated.

