# Supplementary material for: The endothelial plasma membrane lipidome and its remodeling under hyperglycemia: an exploratory study
Source: Front Mol Biosci. 2026 Feb 16;12:1701375. doi: 10.3389/fmolb.2025.1701375 (PMC12950756; doi:10.3389/fmolb.2025.1701375)
Supplement: Supplementary file 1 [file Table2.docx]

Supplemental Table S2. PCA scores calculated for the 33 lipid species that contributed most to the differentiation between normoglycemia and hyperglycemia conditions.

| **Lipid species** | **PC1 (74%)** | **PC2 (12%)** |
| --- | --- | --- |
| PC O-34:2 | -0,1168 | 0,3651 |
| FA 22:4 | -0,1971 | 0,0525 |
| CL 36:2_38:5 | 0,109 | -0,2748 |
| PE P-18:1/18:2 | -0,2002 | -0,065 |
| TG 56:7 | -0,14 | 0,3118 |
| PS 42:6 | -0,2 | -0,0671 |
| PE 42:5 | -0,1994 | -0,0692 |
| CL 34:2_36:3 | -0,0213 | 0,4792 |
| PS 36:3 | -0,1932 | -0,0602 |
| PE 42:6 | -0,1991 | -0,069 |
| PE 38:7 | -0,1991 | -0,0705 |
| SM 44:2;O2 | -0,1817 | 0,0182 |
| PE 42:7 | -0,1986 | -0,0731 |
| PS 38:0 | -0,1974 | -0,1022 |
| PE 32:2 | -0,1908 | -0,0146 |
| TG 54:6 | -0,1727 | 0,124 |
| PC 36:5 | -0,1999 | -0,049 |
| LPC 22:3 | -0,1322 | -0,172 |
| PE 36:5 | -0,1976 | -0,0928 |
| PI 32:1 | -0,1978 | -0,0961 |
| LPE 16:0 | 0,1867 | -0,0569 |
| PC O-30:0 | 0,0131 | -0,2273 |
| PE P-16:0/22:3 | 0,2002 | -0,026 |
| PS 36:4 | -0,197 | -0,096 |
| SM 43:2;O2 | -0,1743 | 0,0331 |
| FA 16:0 | -0,1192 | -0,3356 |
| PE 40:4 | -0,1772 | 0,2048 |
| PS 34:3 | -0,1961 | -0,0945 |
| DG 36:4 | -0,0322 | -0,2637 |
| PE 38:1 | -0,184 | 0,1335 |
| PE P-18:0/22:4 | -0,1889 | 0,069 |
| PE P-16:0/18:2 | -0,1947 | -0,0746 |
| SM 41:2;O2 | -0,1703 | 0,1616 |
