# Supplementary material for: The endothelial plasma membrane lipidome and its remodeling under hyperglycemia: an exploratory study
Source: Front Mol Biosci. 2026 Feb 16;12:1701375. doi: 10.3389/fmolb.2025.1701375 (PMC12950756; doi:10.3389/fmolb.2025.1701375)
Supplement: Supplementary file 3 [file DataSheet1.docx]

**Supplementary Material**

**The Endothelial Plasma Membrane Lipidome and its Remodeling under Hyperglycemia: an exploratory study**

Ana Reis^1*^, Yahya Sohrabi^2^, Lorena Diaz-Sanchez^3^, Ana Rita Dias Araújo^4^, Merle Leffers^2^, Bruno Antonny^4^, Alisa Rudnitskaya^5^, Rui Vitorino^6^, Irundika HK Dias^3*^

**
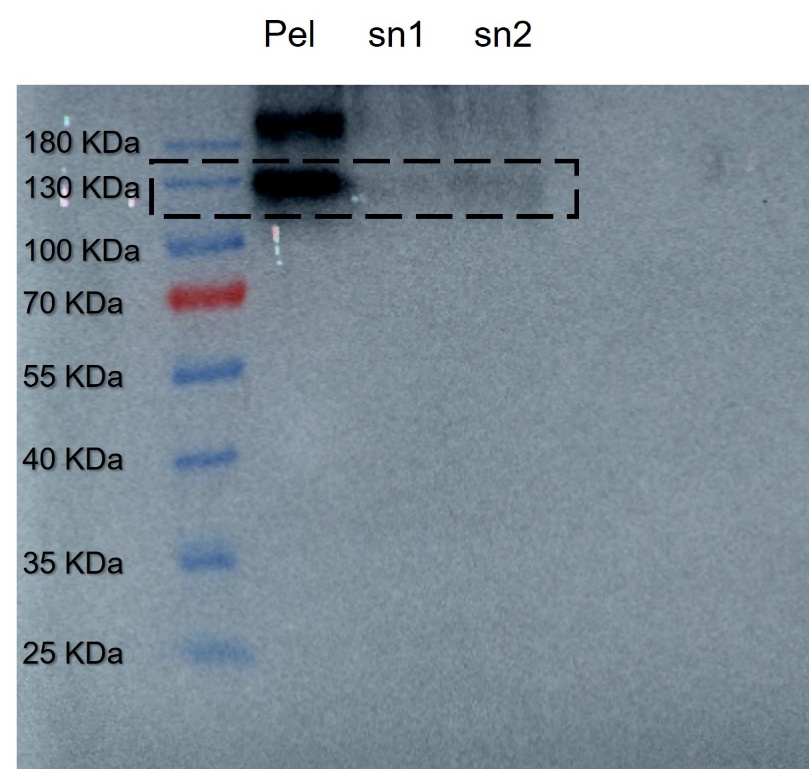
**

**Supplemental Figure S1. Western blot analysis of primary human endothelial giant plasma membrane vesicles (GPMVs)**. Cell-derived GPMVs separated by centrifugation (pellet, Pel) show band in gel (10%) below apparent molecular weight 130kDa representing Na^+^,K^+^-ATPase protein with 110-113 kDa (Lingrel and Kuntzweiler, 1994; Pivovarov et al., 2019). The second band above top marker (180 kDa) may correspond to dimer. Supernatant fractions (sn1 and sn2) collected during centrifugation steps were also included showing very faint bands possibly due to cross-contamination during the pipetting step. Values on the left-hand side lane represent the protein ladder (see Experimental Section for details).


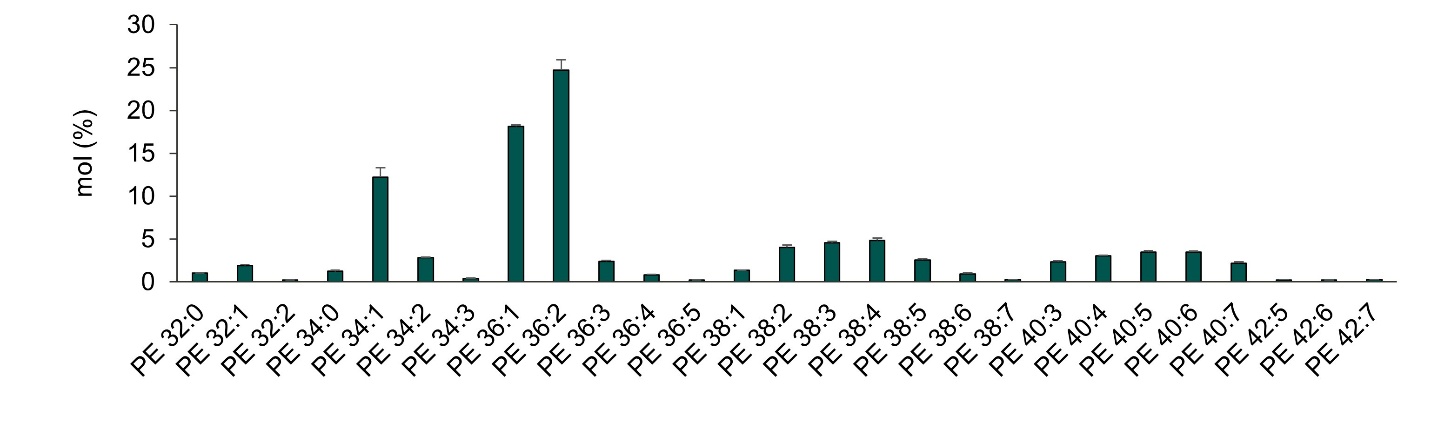


**Supplemental Figure S2. Relative abundance (mol %) of individual PE diacyl species composing endothelial GPMVs.** Predominant diacyl PE correspond to monounsaturated PE (PE 34:1 and PE 36:1) and double unsaturations (PE 36:2). Error bars correspond to standard deviation (SD, *n*=3).

**Supplemental Figure S3**. **Effect of hyperglycemia in the content of lipid species in GPMVs prepared from donors (*n*=3).** A) content (mol %) of lipid species in GPMVs from donors in normo- (light colors) and hyperglycemia (dark colors), B) Variable of Importance in Projection (VIP) index identified by the PLS-DA classification model with the lipid species that contribute most to group discrimination. Color boxes indicate the relative concentrations of the corresponding lipid. The gradient in the right-hand side varies from blue to red and indicate the changes in lipid feature abundance in each group, with blue representing low relative abundance and red representing high relative abundance, C) PCA score plots of discriminative lipid species in normo- (green) and hyperglycemia (orange), with variance explained by the first two components (PC1 and PC2). Boxplots statistical analysis was carried out using a standard two-tailed paired t-test in GraphPad Prism (version 8.4.3., San Diego, CA, USA).


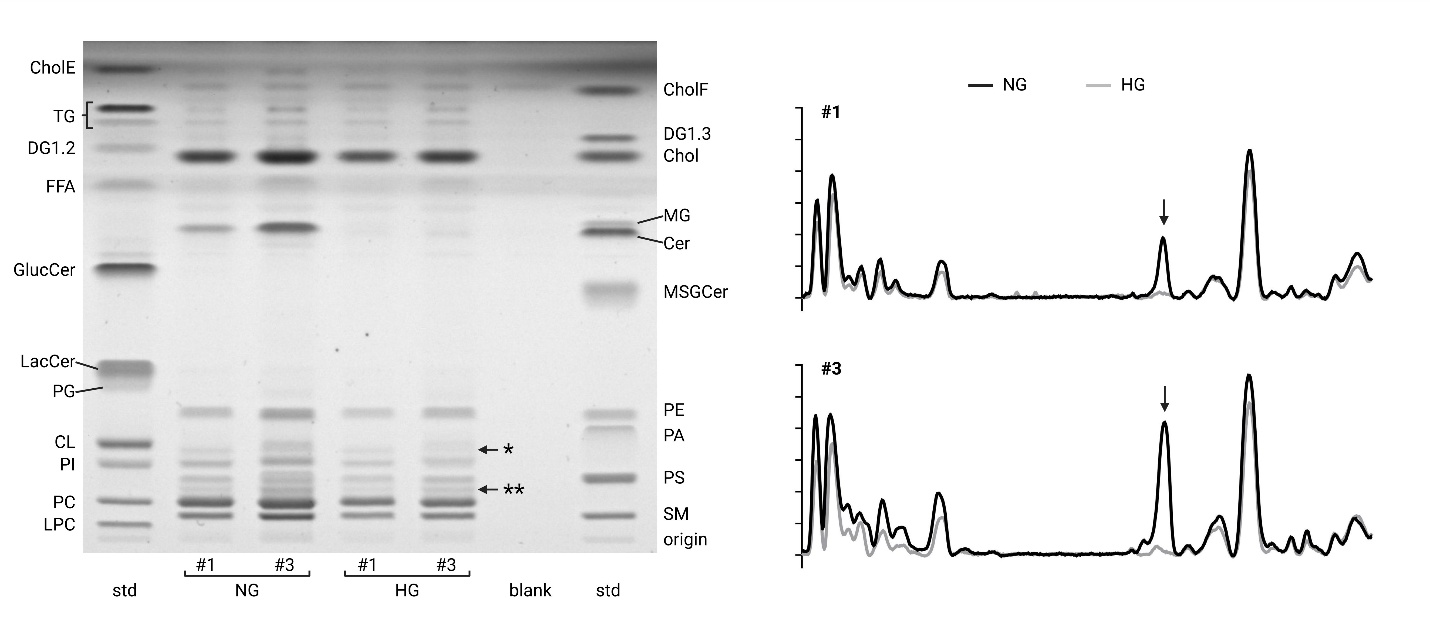


**Supplemental Figure S4.** **HPTLC of GPMVs lipid extracts.** Analysis of GPMVs lipid extracts isolated from HUVECs (donor #1 and #3) grown under normoglycemia (NG) and hyperglycemia conditions (HG). Plates were developed using epi white light. Lanes (from left to right) correspond to biological replicates from donor #1 (lanes 2 and 4) and donor #3 (lanes 3 and 5). Lanes corresponding to the elution of lipid standards (lanes 1 and 7) and blank (lane #6, extraction solvent) are also shown. Chromatograms shown on the right-hand side depict the profile for donor #1 normo- (black line) and hyperglycemia (grey line). Arrows in chromatograms indicate the band showing a marked decrease in its content in hyperglycemia conditions. Standards included in the mixture refer to CholE: cholesteryl ester; CholF: cholesteryl formate; TG: triglycerides; DG1.3: 1,3-diacylglycerides; DG1.2: 1,2-diacylglycerides; Chol: cholesterol; FFA: free fatty acids; MG: monoacylglycerides; Cer: ceramides; GlcCer: glucosyl-ceramide; MSGCer: monosulfo-galactosyl-ceramide (sulfatide); LacCer: lactosyl ceramide; PG: phosphatidylglycerol; PE: phosphatidylethanolamine; PA: phosphatidic acid; CL: cardiolipin; PI: phosphatidylinositol; PS: phosphatidylserine; PC: phosphatidylcholine; LPC: lyso-phosphatidylcholine; SM: sphingomyelin. For additional details on the standards, please see Materials and Methods. Asterisk notation (*) corresponds to the possible contribution of Hex_3_Cer; (**) corresponds to the possible contribution of complex GSL (e.g. gangliosides). Blank corresponds to extraction solvents. Arrow depicts possible depletion of Cer.

References

Lingrel, J.B., & Kuntzweiler, T. (1994). Na+,K+-ATPase. Journal Biological Chemistry. 269, 19659-19662.

Pivovarov, A.S., Calahorro, F., Walker, R.J. (2019) Invertebrate Neuroscience. 19, 1. doi.org/10.1007/s10158-018-0221-7
